# Supplementary material for: Adult Skeletal Age-at-Death Estimation through Deep Random Neural Networks: A New Method and Its Computational Analysis
Source: Biology (Basel). 2022 Mar 30;11(4):532. doi: 10.3390/biology11040532 (PMC9028470; doi:10.3390/biology11040532)
Supplement: Supplementary file 1 [file biology-11-00532-s001.zip › biology-1604784-supplementary.pdf]

biology

Supplementary Materials

Adult Skeletal Age-At-Death Estimation Through Deep Random Neural Networks: A New Method And Its Computational Analysis

David Navega[1, 2,*], Ernesto Costa[3], Eugénia Cunha[1, 2]

1 Centre for Functional Ecology (CEF), Laboratory of Forensic Anthropology, Department of Life Sciences, University of Coimbra, 3000-456 Coimbra, Portugal.

2 National Institute of Legal Medicine and Forensic Sciences, 3000-548 Coimbra, Portugal.
david.s.navega@inmlcf.mj.pt; eugenia.m.cunha@inmlcf.mj.pt.

3 Centre for Informatics and Systems of the University of Coimbra (CISUC), Evolutionary and Complex Systems Group (ECOS), Department of Informatics Engineering, University of Coimbra, 3030-290 Coimbra, Portugal.
ernesto@dei.uc.pt

* Correspondence: dsnavega@gmail.com

Table S1 Scoring system for suture obliteration.

**Stage 0**	[Open or juxtaposed]<br>The sutural segment is characterized by a distinguishable gap between the cranial bones. The sutural gap might be narrow and the bones tightly juxtaposed.
**Stage 1**	[Partially obliterated or totally obliterated]<br>The sutural segment is partially obliterated or totally obliterated. Remnants of the suture might be visible as scattered bony bridges or grooves.

Table S2 List of cranial and palatine suture segment analyzed.

CRS01	Palatine (posterior median)
CRS02L	Palatine (transverse, left)
CRS02R	Palatine (transverse, right)
CRS03	Coronal - Sagittal (pars bregmatica)
CRS04L	Coronal (pars pterica, left)
CRS04R	Coronal (pars pterica, right)
CRS05	Sagittal - Lambdoid (pars lambdica)
CRS06L	Lambdoid (pars asterica, left)
CRS06R	Lambdoid (pars asterica, right)

Table S3 Scoring system for S1-S2 fusion.

**Stage 0**	[Fusing]<br>S1 - S2 fusion is incomplete. On the anterior surface of the sacrum, there is a gap* between the sacral bodies of the S1 and S2.
**Stage 1**	[Fused]<br>The sacral bodies of the S1 and S2 are completely fused. No gap* is visible on the anterior sacral surface.

* The gap seen on the anterior surface of the sacrum should be a discontinuity between the sacral bodies extending for more 10 millimeters in length.

Table S4 Scoring system for vertebral body development and degeneration.

**Stage 0**	[Absence of degenerative changes]<br>a) Incomplete or partially epiphyseal ring fusion. Residual fusion line may be observed on vertebral body. Billows or radiating grooves may also be visible perpendicular to the margin of the vertebral body margin.<br>b) The epiphyseal ring is fully fused forming an elevated border and no degenerative change is observed on the vertebral body margin. Surface is dense and compact.
**Stage 1**	[Transitive stage]<br>The vertebral margin is characterized by small segments where the edge of the margin is sharp but not necessarily lipped. The vertebral body surface is characterized by a flattened aspect. The vertebral ring has a compressed appearance. Microporosities might be visible but usually have a restricted spatial distribution.
**Stage 2**	[Presence of degenerative changes]<br>The vertebra is characterized by its lipped and/or porous aspect. At least one large bony projection protrudes from the body margin (approximately four millimeters or more). The surface of the vertebral body is pitted and irregular.<br>(Vertebrae fused by lipping or ossification and calcification of the vertebral ligaments [i.e., candlewax lesions] should be scored as Stage 2.)

Table S5 List of traits analyzed in the cervical, lumbar, and sacral vertebrae.

Cervical	C3IS	C3 body inferior surface and margin
	C4SS	C4 body superior surface and margin
	C4IS	C4 body inferior surface and margin
	C5SS	C5 body superior surface and margin
	C5IS	C5 body inferior surface and margin
	C6SS	C6 body superior surface and margin
	C6IS	C6 body inferior surface and margin
	C7SS	C7 body superior surface and margin
Lumbar	L1IS	L1 body inferior surface and margin
	L2SS	L2 body superior surface and margin
	L2IS	L2 body inferior surface and margin
	L3SS	L3 body superior surface and margin
	L3IS	L3 body inferior surface and margin
	L4SS	L4 body superior surface and margin
	L4IS	L4 body inferior surface and margin
	L5SS	L5 body superior surface and margin
Sacral	S1SS	S1 body superior surface and margin
	S1S2F	S1-S2 fusion

Table S6 List of traits used to assess joint and musculoskeletal degeneration of the limbs.

Trait		Type	Complex
SC01	Scapula (glenoid fossa)	Joint	Shoulder
HM01	Proximal humerus (head)	Joint	Shoulder
HM02	Proximal humerus (lesser tubercle)	Musculoskeletal	Shoulder
HM03	Proximal humerus (greater tubercle)	Musculoskeletal	Shoulder
HM04	Distal humerus (trochlea and capitulum)	Joint	Elbow
HM05	Distal humerus (medial epicondyle)	Musculoskeletal	Elbow
HM06	Distal humerus (lateral epicondyle)	Musculoskeletal	Elbow
UL01	Proximal ulna (articular facets)	Joint	Elbow
UL02	Proximal ulna (olecranon)	Musculoskeletal	Elbow
RD01	Proximal radius (head)	Joint	Elbow
RD02	Proximal radius (radial tuberosity)	Musculoskeletal	Elbow
OC01	Os coxa (iliac tuberosity)	Musculoskeletal	Hip
OC02	Os coxa (ischial tuberosity)	Musculoskeletal	Hip
OC03	Os coxa (acetabulum)	Joint	Hip
FM01	Proximal femur (head)	Joint	Hip
FM02	Proximal femur (trochanteric fossa)	Musculoskeletal	Hip
FM03	Proximal femur (greater trochanter)	Musculoskeletal	Hip
FM04	Proximal femur (lesser trochanter)	Musculoskeletal	Hip
FM05	Distal femur (condyles)	Joint	Knee
TB01	Proximal tibia (condyles)	Joint	Knee
PT01	Patella (articular facets)	Joint	Knee
PT02	Patella (base)	Musculoskeletal	Knee
CLN01	Patella (calcaneal tuberosity, superior)	Musculoskeletal	Ankle

Table S7 Generic scoring system for joint degeneration traits.

Stage 0	[Absence of degenerative joint changes]<br>Joint margin is smooth and retains normal morphology. Subchondral surface is dense and smooth.
Stage 1	[Presence of degenerative joint changes]<br>Joint margin presents osteophytes that can range from isolated bony edges to large structures on the entire joint margin or most of it rendering it normal contour irregular. Porosities may be present both on the margin and subchondral bone surface but are less common. Bony exostosis may be present on the subchondral surface. The most extreme cases are characterized by eburnation lesions and loss of articular morphology.

Table S8 Generic scoring system for musculoskeletal degeneration traits.

Stage 0	[Absence of degenerative musculoskeletal changes]<br>The surface of the attachment site contour or margin is regular and smooth.
Stage 1	[Presence of degenerative musculoskeletal changes]<br>(One or two conditions can be present)<br>I) The contour of the attachment site is irregular or salient. It manifests as a small bony crest or enthesophyte.<br>II) The bone surface either presents slight irregularities in the form of a diffuse granular texture or more significant types of bone remodeling such as bony excrescences, erosions, or cavitation (large perforations).

Table S9 Stage 1 specific descriptions for selected joint and musculoskeletal degeneration traits.

Trait(s)	Stage 1
SC01	The key aspect is the lipping of the articular margin. Lipping (irregular bony growth) should be considered present if at least one third of the margin is affected.
HM01	The key aspect is the lipping of the articular margin. Lipping is not conspicuous as in the glenoid fossa. In early stages it takes the form of sharp elevated rim that interrupts the flow of the articular surface to para-articular region. The most severe cases form a collar or ring like structure around the humeral head.
HM04	Most common degenerative trait observed is the presence of marginal osteophytes. If present, eburnation usually affects the capitulum.
UL01	The key aspect is the lipping of the articular facets. Usually, the lipping is not so marked as in other joints. Eburnation and other type of surface remodeling are uncommon.
RD01	Marginal lipping and porosities both on the surface and margin are the most common degenerative aspects. In some cases, the bone surface seems thinned out (loss density).
OC03	Osteophytic growth of the posterior *cornum* is common evidence of early degenerative changes of the acetabulum. Osteophytic growth of inner margin can obliterate the acetabular fossa. Lunate surface remodeling is uncommon but in severe cases eburnation can be present. The acetabular fossa may present textural changes expressed as porosities, bony growths, and granularity.
FM01	Marginal osteophytosis and surface remodeling such as the formation of bone nodules and irregularities of the contour of *fovea capitis* are common. In extreme cases, osteophytic activity creates an osteophytic ring around the femoral head. In some cases, the bone surface and the margin appear thinned out (loss of density).
FM05	Surface porosity and marginal lipping are the most common degenerative traits observed. Eburnation is observed in extreme cases of joint degeneration.
TB01	Surface porosity and lipping are the most common degenerative traits observed. Eburnation is observed in extreme cases of joint degeneration.
PT01	Surface porosity and lipping are the most common degenerative traits observed. Eburnation is observed in extreme cases of joint degeneration.

Table S10 Scoring system for clavice age-related traits.

Trait(s)		Stage	Description
CLV01	Sternal end	0	Epiphyseal union at the sternal end of the clavicle is incomplete (nonunion with or without epiphysis) or partial.
		1	Epiphyseal fusion is complete. The sternal surface has smooth to finely granular texture. The surface contour maintains a normal appearance without osteophytic irregularities. Porosities (micro or macropores) are not a characterizing feature, when they occur spatial distribution is limited (less than one-third of the surface).
		2	Sternal end is characterized by a coarsely granular texture (bone exostosis). Porosity (micro and macropores) occur in more than half of the surface. The surface contour may present an irregular profile due to osteophytic activity.
CLV02	Acromial end	0	Bone surface is smooth or finely granular.
		1	Acromial end is characterized by the presence of a coalescent porosity pattern of macropores. Bone surface appear thinned out and trabecular bone may be exposed.

Table S11 Scoring system for the first rib age-related traits.

Trait(s)		Stage	Description
RB101	Costal face	0	Costal face presents a narrow profile. The costal surface has flat profile characterized by the presence of transverse ridges or a smooth texture. The periarticular bone surface is smooth.
		1	The topography and texture of the costal face are characterized by an increasing concavity and cribriform pattern. The margins are slightly projected with scalloped edges. At the anterosuperior aspect of the margin, elongated spicules may form a rugged shaft around the costal face.
		2	Costal face is characterized by extensive ossification of costochondral interface. Ossification surrounds most of the costal face and may form a hollow shell around it. Periarticular region is rugged. Sternocostal fusion may occur.
RB102	Tubercule	0	Tubercle is characterized by rounded and smooth articular margins. The periarticular region is smooth.
		1	The tubercle facet is characterized by a coarsely granular texture. Porosities may occur in the articular surface. Lipping of the articular margins may occur. The periarticular region is rugose.

Table S12 Scoring system for the pubic symphysis age-related traits.

Trait(s)		Stage	Description
PSY01	Rim	0	Symphyseal rim is incomplete. In the early stage of rim formation there is a continuum between symphyseal face and adjacent structures (pubic tubercule, pubic ramus). Rim formation later evolves from an early rampart that forms on the cranial, dorsal, caudal and ventral portions of the rim.
		1	Symphyseal rim is complete. An elevated bony rim delineates the symphyseal face demarcating it from adjacent structures such as the pubic tubercle. In some specimens, the superior segment of the ventral margin might not fully form into a rim after early formation of the ventral rampart. This condition, ventral hiatus, should not be confused with incomplete rim formation (Stage 0) or rim breakdown (Stage 2).
		2	Symphyseal rim is breaking down. Breakdown is characterized by lipping and erosion (porosity, pitting) of the ventral and dorsal margins. Breakdown of the symphyseal rim is usually associated with ligamental outgrowths and a bony plaque on the ventral and dorsal surface of the pubic bone.
PSY02	Topography	0	Symphyseal topography is characterized by a billowing pattern (alternating ridge and furrows). In early stages this pattern is very sharp but as symphyseal face flattens (Stage 1), it becomes shallow and residual (usually one patch defined by two consecutives flattened ridges).
		1	Symphyseal surface is flat and homogeneous.
		2	Symphyseal topography is irregular and depressed in relation to the symphyseal rim.
PSY03	Texture	0	Symphyseal texture is smooth to finely grained and have dense aspect.
		1	Symphyseal texture is coarsely granular yet homogeneous. Scattered porosities (micropores) may occur throughout the surface.
		2	Symphyseal texture appears eroded and is characterized by clustered porosities and irregular bony formations. Texture appears to be less dense.

Table S13 Scoring system for the sacral auricular age-related traits.

Trait(s)		Stage	Description
SAS01	Texture	0	Surface is characterized by a homogeneous smooth to finely granular texture. Bone surface has a dense and compact aspect. A structured relief pattern characterized by a shallow billows or *striae* from early development stage may remain visible (residually). No porosities are observed.
		1	Surface is characterized by coarsely granular texture. Porosity occurs throughout the surface in a scattered or clustered pattern (both macro and/or micropores).
SAS02	Margin	0	The margin of the auricular surface is smooth and well defined.
		1	The contour of the auricular surface is marked by several irregularities. The margin of the surface is sharped (lipped), more commonly in the anterior and inferior apices.

Table S14 Scoring system for the iliac auricular age-related traits.

**Trait(s)**		**Stage**	**Description**
IAS01	Texture	0	Surface is characterized by a homogeneous smooth to finely granular texture. Bone surface has a dense and compact aspect. A structured relief pattern characterized by a shallow billows or *striae* from early development stage may remain visible (residually). No porosities are observed.
		1	Surface is transitioning from a finely granular to coarsely granular texture. Small exostoses may occur but are not a dominant textural element. Porosity (micropores) occurs throughout the surface in a scattered pattern.
		2	Surface is characterized by an irregular granular texture. Porosity is the dominant textural element—clustered distribution and presence of macropores. In overall, the surface has an irregular and eroded aspect.
IAS02	Margin	0	The margin of the auricular surface is smooth and well defined.
		1	The contour of the auricular surface is marked by several irregularities. The margin of the surface is sharped (lipped), more commonly in the anterior and inferior apices.

Table S15 Scoring system for the acetabular age-related traits.

Trait(s)		Stage	Description
ACT01	Rim	0	Acetabular rim is dense and smooth to the touch. The edge along the rim presents a rounded profile with no significant porosity. The area adjacent to the acetabular rim has no significant porosity and its surface is also dense and smooth.
		1	New bone formation, osteophytic activity, is visible on some regions of the rim. It is manifested as a small (approximately one millimeter) osteophytic crest along most of the rim or as a crest with a higher profile (approximately two to four millimeters) only on a portion of the rim. The osteophytic crest is usually dense with no porosity on newly formed bone. The rim is not smooth to the touch and macroporosity may occur. Adjacent areas of the rim, such as the posterior wall of acetabulum and the region below the anterior inferior iliac spine may present porosities and textural changes that render the bone surface rough to the touch.
		2	The acetabular rim has an irregular profile as a by-product of osteophytic and osteolytic processes. A high profiled osteophytic crest (superior to four millimeters) is usually present at this stage. Such osteophytic cresting is accompanied by porotic changes on the newly formed bone which, in overall, renders the acetabulum a fragile and eroded aspect. Porosity and new bone formation can invade the lunate surface (usually below the anterior inferior iliac spine or around the ilium-ischium intersection).
ACT02	Posterior horn	0	The apex is round and smooth to the touch and no bony spur is visible.
		1	The apex is rough and sharp to the touch, and a small spicule or spur can be felt (approximately two millimeters). It is circumscribed to a small part of the horn edge.
		2	A conspicuous bony spur (superior to three millimeters) is present. This proliferative feature is variable in its magnitude and extent. In extreme cases bone proliferation occupies the acetabular notch and may completely cross it or form a bony bridge. This stage usually co-occurs with more advanced stages of morphological degeneration of the acetabular rim.
ACT03	Fossa	0	The acetabular fossa center is very dense with a smooth texture. The outer edge of the acetabular fossa, along the inner border of the lunate surface, has a smooth edge with no osteophytic activity.
		1	The outer edge of acetabular fossa presents early degenerative changes. The edge is rough to the touch and the osteophytic structure can affect only a portion or the entire edge but is usually small (approximately one to three millimeters). The central region of the fossa may present a rougher bone surface (Stage 0) characterized by an increase in textural irregularities and porosities. Yet, the fossa does not have a fragile and irregular aspect due to extensive bone remodeling.
		2	The central region of the acetabular fossa has lost bone density and structural consistency. Porosity is a key aspect at this stage. Trabecular bone might be exposed due to extensive osteolytic and osteophytic processes. Porotic lesions have an irregular and sharp perimeter. Compared to the previous stage, osteophytic cresting, along with the outer edge of the fossa, is more pronounced both in extent and magnitude. In extreme cases, an osteophytic visor obliterates partly the fossa.
